# Finding of the Low Molecular Weight Inhibitors of Resuscitation Promoting Factor Enzymatic and Resuscitation Activity

**DOI:** 10.1371/journal.pone.0008174

**Published:** 2009-12-16

**Authors:** Galina R. Demina, Vadim A. Makarov, Vadim D. Nikitushkin, Olga B. Ryabova, Galina N. Vostroknutova, Elena G. Salina, Margarita O. Shleeva, Anna V. Goncharenko, Arseny S. Kaprelyants

**Affiliations:** A. N. Bach Institute of Biochemistry, RAS, Moscow, Russia; University of Cape Town, South Africa

## Abstract

**Background:**

Resuscitation promoting factors (RPF) are secreted proteins involved in reactivation of dormant actinobacteria, including *Mycobacterium tuberculosis*. They have been considered as prospective targets for the development of new anti-tuberculosis drugs preventing reactivation of dormant tubercle bacilli, generally associated with latent tuberculosis. However, no inhibitors of Rpf activity have been reported so far. The goal of this study was to find low molecular weight compounds inhibiting the enzymatic and biological activities of Rpfs.

**Methodology/Principal Findings:**

Here we describe a novel class of 2-nitrophenylthiocyanates (NPT) compounds that inhibit muralytic activity of Rpfs with IC_50_ 1–7 µg/ml. Fluorescence studies revealed interaction of active NPTs with the internal regions of the Rpf molecule. Candidate inhibitors of Rpf enzymatic activity showed a bacteriostatic effect on growth of *Micrococcus luteus* (in which Rpf is essential for growth protein) at concentrations close to IC_50_. The candidate compounds suppressed resuscitation of dormant (“non-culturable”) cells of *M. smegmatis* at 1 µg/ml or delayed resuscitation of dormant *M. tuberculosis* obtained in laboratory conditions at 10 µg/ml. However, they did not inhibit growth of active mycobacteria under these concentrations.

**Conclusions/Significance:**

NPT are the first example of low molecular weight compounds that inhibit the enzymatic and biological activities of Rpf proteins.

## Introduction

Tuberculosis (TB) still kills millions of people around the Globe. Moreover, *Mycobacterium tuberculosis* has been suggested to persist in a dormant state in approximately 2 billion of people [1] (*Dormancy* is a reversible physiological state of the bacteria characterized by low metabolic activity, in which cells can persist for extended periods without division). Due to reasons that are not fully clear, such latent infection can reactivate at any time causing active tuberculosis [2] (*Latency* is a clinical state of asymptomatic, chronic infection). Importantly, dormant mycobacteria are less susceptible to antibiotics which are normally used for treatment of active tuberculosis. Therefore, finding of new compounds with potential activity against latent forms of tuberculosis is an urgent task for the scientific community. Ideally, such compounds should sterilize dormant *M. tuberculosis* in the host environment or block its reactivation.

The discovery of Rpfs (resuscitation promoting factor), a family of proteins involved in the control of dormancy and “non-culturability” of mycobacteria and related organisms, has provided a promising opportunity to explore new strategies on targeting *M. tuberculosis* persistent organisms associated with latent infections. *M. tuberculosis* contains five *rpf*-like genes which products, RpfA-E expressed as recombinant proteins in *E. coli* stimulated replication and resuscitation of mycobacterial cells [3], [4]. The importance of the Rpf proteins for resuscitation of dormant (“non-culturable”) M. *tuberculosis* cells *in vitro* and for growth of viable cells *in vivo* was also confirmed in the study of the Rpf knockout mutants [5], [6].Despite these mutants were defective for reactivation from chronic tuberculosis [7], [8] the possible significance of Rpfs for establishing and maintenance of latent tuberculosis has yet to be clarified. Therefore, Rpfs represent attractive targets for development of new drugs preventing resuscitation of dormant *M. tuberculosis*. Such drugs will have a great potential for prophylaxis of latent TB reactivation, provoked by the application of anti-TNF antibodies therapy [9]. However, until recently, no substances inhibiting the biological activity of Rpf have been reported.

Despite the progress in the Rpf research, the mechanisms of its biological activity remain unclear. Recent findings suggest that Rpfs are probably involved in the remodelling of bacterial cell envelope [10], [11], [12]. According to NMR [13] and X – ray diffraction [14] studies of the conserved domain of Rpf is structurally close to the c-type lysozyme. However, functionally it is more similar to lytic transglycosylases [12]. Several experimental results confirmed that Rpf possesses cell wall hydrolysing activity. Moreover, a correlation between this enzymatic activity and the resuscitation activity of Rpf has been found [15]. In particular, the ability of recombinant Rpf protein to hydrolyze the artificial lysozyme substrate, 4-methylumbelliferyl-β-D-N,N′,N″-triacetylchitotrioside (4-MUF-3-NAG) [15], [16] opened a possibility for the fast screening of low molecular weight compounds which could serve as inhibitors of the Rpf proteins. In this paper we report on several nitrophenylthiocyanates (NPT) with anti-Rpf activity.

## Materials and Methods

### Chemistry

Melting points were determined according to the BP procedure and are uncorrected (Electrothermal 9001, GB). If analyses are indicated only by the symbols of the elements, analytical results are within ± 0.3% of the theoretical values (Carlo-Erba 5500, Italy). NMR spectra were determined with a Varian Unity Plus 400 (USA). Shifts for ^1^H NMR are reported in ppm downfield from TMS (σ). Mass spectra were obtained using a Finnigan SSQ-700 (USA) instrument with direct inject. Silicagel 60 F_254_ aluminium sheets (Merck Co, Germany) were used for all analytical thin layer chromatography (TLC). All chemicals were purchased from Alfa Chemicals (Lancashire, England) including starting otho-chloronitroaryles for synthesis target compounds (III), (IV), (V), (VI), (VIII), (IX), (X).

4-chloro-6-methoxy-5-nitropyrimidine was synthesised according to [17].

#### General procedure for 2-thiocyanatonitroaryles synthesis (Figure 1)

A suspension of 2-chloronitroaryles (4.00 mmol) and 1,5-dichloroantraquinone (3 mg, 0.011 mmol) in ethanol (25 ml) was treated by 10 ml ethanol solution of KSCN (0.58 g, 6.00 mmol) and heated at 50°C for 2–6 h. After cooling the reaction mixture was dissolved in 100 ml of cold water and light yellow precipitate was collected by filtration. The solid residue was twice crystallised from suitable solvent to obtain 2-thiocyanatonitroaryles with >96% purity (yield 35–55%).

**Figure 1:**
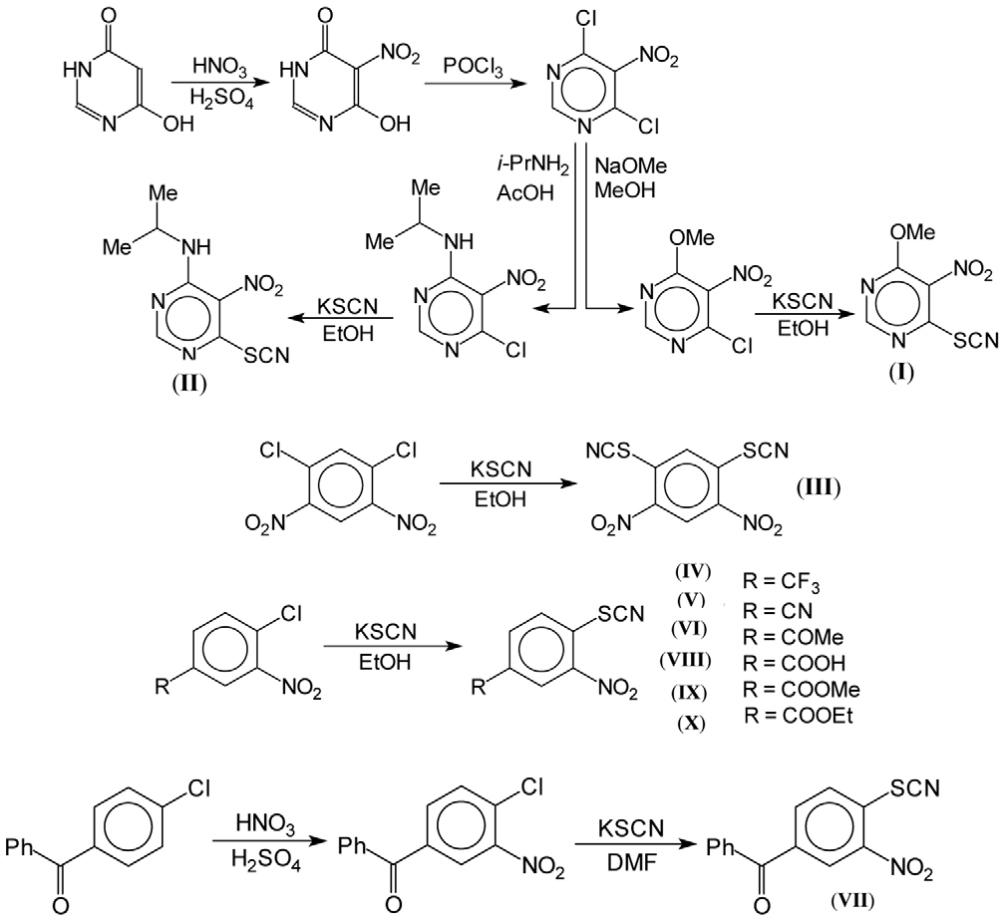
Schematic representation of 2-thiocyanatonitroaryles (I)–(X) synthesis.

#### 4-Methoxy-5-nitro-6-thiocyanatopyrimidine (I)

Suspension of 1.94 g (1.0 mmol) of 4,6-dichloro-5-nitropyrimidine in 20 ml methanol was treated by methanol solution of MeONa (prepared in advance from 0.23 g (1.0 mmol) of natrium and 10 ml of methanol) at 0°C. Reaction mixture was stored for 2 h at room temperature and mixed with 100 ml of cold water. White crystalline solid of 4-chloro-6-methoxy-5-nitropyrimidine was filtered off and crystallised from ethanol (mp. 54–56°C). Follow reaction according “General procedure for 2-thiocyanatonitroaryles synthesis” leads to the target compound (I). Mp 128–31°C (ethanol). m/z^+.^ 212. ^1^H NMR (DMSO) σ 9.25 (1H, s, CH) and 4.17 (3H, s, CH_3_). Anal. (C_6_H_4_N_4_O_3_S), C,H,N,S.

#### 4-Isopropylamino-5-nitro-6-thiocyanatopyrimidine (II)

Solution of 1.94 g (1.0 mmol) of 4,6-dichloro-5-nitropyrimidine in 20 ml dioxane was treated by water solution of isopropylamine acetate (prepared in advance from 1.20 g (2.0 mmol) of isopropylamine and excess of acetic acid in 5 ml of water). Reaction mixture was stored for 2 h at room temperature and mixed with 100 ml of cold water. Light yellow crystalline solid of 4-chloro-6-isopropylamine-5-nitropyrimidine was filtered out and crystallised from ethanol (mp. 96–98°C). Follow reaction according “General procedure for 2-thiocyanatonitroaryles synthesis” leads to the target compound (II). Mp 143–5°C (ethanol). m/z^+.^ 239. ^1^H NMR (DMSO) σ 8.43 (1H, s, CH), 4.86 (1H, m, CH(CH_3_)_2_) and 1.37 (6H, s, 2CH_3_). Anal. (C_8_H_9_N_5_O_2_S), C,H,N,S.

#### 1,5-Dinitro-2,4-dithiocyanatobenzene (III)

Usage of 1,5-dinitro-2,4-dichlorobenzene according “General procedure for 2-thiocyanatonitroaryles synthesis” leads to the target compound (III). Mp 78-80°C (DMF/water). m/z^+.^ 282. ^1^H NMR (DMSO) σ 9.66 (1H, s, CH) and 8.61 (1H, s, CH). Anal. (C_8_H_2_N_4_O_4_S_3_), C,H,N,S.

#### 2-Nitro-4-(trifluoromethyl)-phenylthiocyanate (IV)

Usage 4-chloro-3-nitrobenzotrifluoride according “General procedure for 2-thiocyanatonitroaryles synthesis” leads to the target compound (IV). Mp 78–81°C (ethanol). m/z^+.^ 248. ^1^H NMR (DMSO) σ 8.51 (1H, s, CH), 8.29 (1H, d, CH) and 7.84 (1H, d, CH). Anal. (C_8_H_3_F_3_N_2_O_2_S), C,H,N,S.

#### 3-Nitro-4-thiocyanobenzonitryl (V)

Usage 4-chloro-3-nitrobenzonitrile according “General procedure for 2-thiocyanatonitroaryles synthesis” leads to the target compound (V). Mp 143-5°C (ethanol). m/z^+.^ 205. ^1^H NMR (DMSO) σ 8.40 (1H, s, CH), 8.26 (1H, d, CH) and 7.90 (1H, d, CH). Anal. (C_8_H_3_N_3_O_2_S), C,H,N,S.

#### 4-Acetyl-2-nitrophenyl thiocyanate (VI)

Usage 4′-chloro-3′-nitroacetophenone according “General procedure for 2-thiocyanatonitroaryles synthesis” leads to the target compound (VI). Mp 96-8°C (ethanol). m/z^+.^ 222. ^1^H NMR (DMSO) σ 8.69 (1H, s, CH), 8.09 (1H, d, CH), 7.85 (1H, d, CH) and 2.57 (3H, s, CH_3_). Anal. (C_9_H_6_N_2_O_3_S), C,H,N,S.

#### 4-Benzoyl-2-nitrophenyl thiocyanate (VII)

Solution of 4.35 g (2.0 mmol) 4-chlorobenzophenone in 6 ml of sulfuric acid was treated by mixture of 2 ml of nitric acid and 2 ml of sulfuric acid at 0°C. Reaction mixture was warmed up to the room temperature, heated at 65°C for 2 h and put on 100 g of crashed ice. White solid of 4-chloro-3-nitrobenzophenone was collected, dried and crystallized from hexane (mp 104–6°C). Follow reaction according “General procedure for 2-thiocyanatonitroaryles synthesis” leads to the target compound (VII). Mp 132–5°C (ethyl acetate). m/z^+.^ 284. ^1^H NMR (DMSO) σ 8.58 (1H, s, CH), 8.11 (1H, d, CH), 7.93 (1H, d, CH), 7.67 and 7.41 (5H, m, C_6_H_5_). Anal. (C_14_H_8_N_2_O_3_S), C,H,N,S.

#### 3-Nitro-4-thiocyanatobenzoic acid (VIII)

Usage 4-chloro-3-nitrobenzoic acid according “General procedure for 2-thiocyanatonitroaryles synthesis” leads to the target compound (VIII). mp 220-4°C (ethanol/water). m/z^+.^ 224. ^1^H NMR (DMSO) σ 8.56 (1H, s, CH), 8.18 (1H, d, CH) and 7.87 (1H, d, CH). Anal. (C_8_H_4_N_2_O_4_S), C,H,N,S.

*3-Nitro-4-thiocyanatobenzoic acid methyl ester* (IX) and *3-nitro-4-thiocyanatobenzoic acid ethyl ester* (X) were synthesized according [18]. The physico-chemical properties of compounds (IX) and (X) were identical to the reported ones.

### Bacterial Growth

*Micrococcus luteus*, Fleming strain 2665, NCIMB13267 was grown in Nutrient Broth (Hi Media) on an orbital shaker at 30°C. *Mycobacterium smegmatis* mc^2^155 was grown in Sauton's medium on an orbital shaker at 37°C. *M. tuberculosis* H37Rv was grown under agitation at 37°C (200 rpm), in Sauton's medium supplemented by ADC with 0.05% w/v Tween 80 [19] or in 7H9 liquid supplemented with 10% v/v of OADC (BD) and 0.05% w/v of Tween 80. Specially modified media (see below) were applied for generation of “non-cultutrable” mycobacteria.

### MIC Determination

Cells of *M. luteus* were grown to the stationary phase in the rich medium (NB, Himedia) for 48 h. Cultures were washed twice by centrifugation and diluted to 10^5^ cells/ml by the same medium. Diluted cells were distributed on 96 well plates (250 µl per well) and incubated in the Multiskan Analyzer (Thermo, Finland) with 620 nm filter at 30°C for 24 h with shaking. Prior the incubation, the inhibitors, freshly dissolved in DMSO (1 mg/ml), were added in 0.5–10 µg/ml concentration range to the appropriate wells in triplicates. The culture of *M. luteus* without inhibitors served as a control. Bacterial growth was followed by measurement of optical density at 620 nm. Cells of *M. smegmatis* grew in NB medium to the stationary phase for 24–26 h and were diluted to 10^5^ cells/ml by the same medium containing 0.05% w/v Tween-80. Cell growth with and without inhibitors was detected at 37°C in the Multiskan Analyzer, similarly to *M. luteus* experimental conditions. *M. tuberculosis* grew in Sauton's medium supplemented by ADC in the presence of 0.05% Tween-80 for 8 days. Cells were inoculated in 2 ml of fresh medium at ca 10^6^ cells/ml in test tubes. Inhibitors were added at 1-25 µg/ml in triplicates. Test tubes were incubated without shaking at 37°C OD_600_ was measured periodically, using Eppendopf Biophotometer for 14 days.

### Formation of “Non-Culturable” Cells of M. smegmatis and M. tuberculosis

*M. smegmatis* mc^2^155 harbouring the pAGR plasmid with the *rpf* gene was used to produce “non-culturable” (NC) cells in the stationary phase as described previously [20]. *M. tuberculosis* “non-culturable” cells [21] were established after the prolonged storage (4–5 months) of stationary cultures after cell growth in the modified Sauton's medium without K^+^ for 50 days [22].

### Resuscitation of “Non-Culturable” Cells of M. smegmatis and M. tuberculosis

Resuscitation and most probable number (MPN) assays of *M. smegmatis* were performed in 48-well plastic plates (Corning) containing 1 ml of Sauton's medium diluted 1∶1 with 0,6% (w/v) glycerol in water. Some wells contained inhibitors at 0.1–25 µg/ml. All wells were supplemented with 0.05%(w/v) yeast extract (LabM). Appropriate serial dilutions of the starved *M. smegmatis* cells (100 µl) were added to each well. Plates were incubated at 37°C with agitation at 150 rpm for 10–14 days. Wells with visible bacterial growth were counted as positive, and MPN values were calculated using standard statistical methods [23]. The identity of cells was verified by microscopy using a Nikon microscope (Japan).

Similar procedure was applied for resuscitation of *M. tuberculosis* cells, save that the diluted Sauton's medium was supplemented by 10% v/v ADC, and cell dilutions were prepared in screw capped test tubes which were kept standing at 37°C for 2 months.

### Isolating and Purification of Recombinant Rpf Proteins

The truncated form of *M.luteus* Rpf (RpfSm) was used for the analysis. The truncated protein contained conserved Rpf domain and additional 20 amino acids of the gene product, corresponding to the following amino acid sequence:

ATVDTWDRLAECESNGTWDINTGNGFYGGVQFTLSSWQAVGGEGYPHQASKAEQIKRAEILQDLQGWGAWPLCSQKLGLTQADADAGDVDATE. The truncated gene was amplified by PCR from the pET-19b-Rpf described before [24], using a T7 promoter primer CGCGAAATTAATACGACTCACTAT and a reverse primer: CGACGGATCCTCACTCGGTGGCGTCACGT (BamH1 restriction cite is underlined). After denaturation for 5 min at 94°C, samples were subjected to 2 cycles of: 30 s at 94°C, 30 s at 58°C, 60 s at 72°C, followed by 2 cycles of: 30 s at 94°C, 30 s at 56°C, 60 s at 72°C and 25 cycles of: 30 s at 94°C, 30 s at 54°C, 60 s at 72°C, followed by 5 min at 72°C. The purified PCR product was digested with XbaI and BamH1, purified, ligated into pET19b vector and established in *E. coli* DH5α. The construct containing the truncated *rpf* gene (*RpfSm*) was sequenced and transformed in *E. coli* HSM174 (DE3). RpfSm was purified from 350 ml cultures of the *E. coli* producer strain, grown at 37°C in the rich medium (Hi Media) containing 100 µg/ml ampicillin to optical density OD_600 nm_ 0.65–0.8. After induction with 1 mM IPTG, growth continued for 2 h at room temperature. Cells were collected by centrifugation at 3000 *g* for 15 min and frozen in the binding buffer (BB) (20 mM Tris-HCl pH 8.0; 0.5 M NaCl; 5 mM imidazole). After thawing out, RNAase and DNAase, both at concentrations of 10 µg/ml were added in the presence of 10 MM MgSO_4_, followed by addition of 8 M urea. After sonication, the crude extract was centrifuged at 6000 *g* for 30 min to remove cell debris, and supernatant was applied to the Ni^2+^-chelation column (V = 2 ml) («Sigma», Germany), equilibrated with BB. The Biological LP system («Biorad») was employed for the elution and refolding of the protein on the Ni-NTA column: first, the series of washing steps, with the BB contained 8 M urea, were applied. The second (refolding step) was the application of BB with urea concentration declining linearly from 8 M to zero. The protein was eluted by linear gradient of imidazole from 5 mM to 500 mM. Protein was collected in the range of imidazole concentrations of ∼250 mM in a total volume of 4–5 ml. Dialysis of Rpf contained fraction (30–50 µg/ml) was performed against 50 mM citric acid-sodium citrate buffer, pH 6.0. The protein was stored at +4°C for one week without significant loss of the activity.

_ΔDUF_RpfB was expressed and purified under native conditions according to [25] save that incubation with IPTG was 2 h at room temperature. Final concentration of the protein was 150–250 µg/ml. Dialysis step and storage were similar to RpfSm.

### Muralytic Activity of Rpf

To determine a muralytic activity of the recombinant Rpfs', 4-methylumbelliferyl-β-D-N, N′, N″-triacetylchitotrioside (4-MUF-3-NAG) (“Sigma”, Germany) was applied. 6 µM of 4-MUF-3-NAG was mixed with the proteins (1–10 µg/ml) in 100 mM citric acid-sodium citrate buffer, pH 6.0 with 0.5 mM MgSO_4_ in total volume of 0.4 ml. A number of experiments were performed in the presence of NPT compounds, in concentration range of 0.5–10 µg/ml. After 3 hours of incubation at 37°C, 2 µl of 10 M NaOH was added and the samples were frozen and kept at −20°C. The kinetic analysis of Rpf activity and inhibition was performed using the range of concentrations of the substrate from 3 to 24 µM and the inhibitor (VII) from 0.5 to10 µg/ml. The intensity of fluorescence was detected by the RF-5301PC fluorimeter (“SHIMADZU”, Japan) using excitation wavelength of 364 nm and a read-out of 448 nm.

### CD Spectra

CD analysis of Rpf proteins was performed in a buffer −20 mM phosphate, 50 mM sodium chloride, pH 7.0. The traces of imidazole in the protein were removed by extensive dialysis prior to the CD spectra recording. Measurements were performed using Jasco – 720 automatic recording spectrophotometer; 1 mm path length quvette was used; slit width 1.5 nm; time constant 3 sec. Experiment was performed at 20°C. Inhibitors were used as acetonitrile solution (1 mg/ml).

### Fluorescence Quenching Measurements

The steady-state fluorescence excitation and emission spectra of _ΔDUF_Rpf B were recorded at room temperature using the RF-5301PC fluorimeter (“SHIMADZU”, Japan) in 0.3×1 cm path length quartz quvette with slit width 1.5 nm for excitation and 3 nm for emission channels. Proteins (40–50 µg/ml) were dissolved in 50 mM phosphate buffer, pH 7.5. The fluorescence emission spectra were measured under excitation wavelength at 280 nm. Fluorescence quenching measurements were analysed using the Stern-Volmer equation [26]:
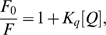
(1)and the modified form of the equation for the heterogeneous type of quenching:
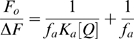
(2)where *F_0_* and *F* are the relative fluorescence intensities in the absence and presence of quencher, respectively, [Q]- concentration of quencher and *K_q_* is the Stern – Volmer quenching constant.

## Results

### Enzymatic Activity of Rpf Is Inhibited by NPT Compounds

Hydrolytic enzymes play a key role for spore germination in many microorganisms [27], [28]. Recently synthesized a family of nitrophenylthiocyanates (NPT), which strongly suppressed germination of fungal spores (unpublished results ) and therefore could be considered as promising inhibitors of cell wall hydrolases, including lytic transglycosylases, a class of enzymes structurally similar to Rpf which are known to be first activated during endospore germination [27] and chitinase involved in fungi spore germination [28]. Therefore, the effect of the influence the NPT compounds (Figure 1) on the enzymatic and biological activities of Rpfs was studied. We first tested the effect of compounds on Rpf muralytic activity judging by the hydrolysis of the artificial fluorogenic substrate, 4-MUF-3-NAG, previously applied to analyze the activity of the recombinant Rpf of *M. luteus* [15]. The Rpf proteins are generally very unstable and inactivate soon after purification [15]. To solve this problem we used two different recombinant Rpf proteins that possess higher stability, solubility and retain activity for longer period of time. Firstly, a truncated form of *M. luteus* Rpf lacking the last 89 amino acid residues of the C-terminal part (Rpf Sm) and secondly, recently described truncated form of Rpf B (_ΔDUF_ RpfB) [14]. Both proteins were able to hydrolyze 4-MUF-3-NAG with the maximum specific activity at 7.6±3.3 nmol/mg·h and 2.5±1.4 nmol/mg·h for RpfSm and _ΔDUF_RpfB correspondingly. We found that four out of five of the initially tested compounds (I)-(IV) and (IX) inhibited muralytic activities of both proteins in the 1–7 µg/ml concentration range (Table 1). The most potent compound for _ΔDUF_RpfB, (IX), was used for synthesis of derivative compounds (V-VIII, X). Apart of negatively charged compound (VIII), the derivative compounds displayed inhibitory activity on the Rpf- mediated MUF-3-NAG hydrolysis (VIII) (Table 1). The Lineweaver-Burk plot revealed non-competitive inhibition of Rpf-Sm muralytic activity by the compound (VII) (Figure S1).

**Table 1:** Influence of NPT compounds on growth of some actinobacteria and on muralytic activity of Rpfs.

Compound	Chemical name	MIC *M. luteus*, µg/ml	4-MUF-3-NAG hydrolysis IC_50_ µg/ml	
			*Rpf Sm*	*Δ_DUF_Rpf B*
I	4-Methoxy-5-nitro-6-thiocyanatopyrimidine	NI	5.3±1.7	ND
II	4-Isopropylamino-5-nitro-6-thiocyanatopyrimidine	NI	NI	NI
III	1,5-Dinitro-2,4-dithiocyanatobenzene	5	1.2±0.7	0.45±0.15
IV	2-Nitro-4-(trifluoromethyl)phenyl thiocyanate	5	5.3±1.4	ND
V	3-Nitro-4-thiocyanobenzonitryl	1–5	5.2±1.1	4.6±2.6
VI	4-Acetyl-2-nitrophenyl thiocyanate	5	2.2±1.3	1.3±0.7
VII	4-Benzoyl-2-nitrophenyl thiocyanate	1–5	1.9±0.9	1.5±1.3
VIII	3-Nitro-4-thiocyanatobenzoic acid	NI	NI	NI
IX	3-Nitro-4-thiocyanatobenzoic acid methyl ester	5–10	5.6±1.5	0.5±0.1
X	3-Nitro-4-thiocyanato-benzoic acid ethyl ester	10	2.6±1.1	ND

MIC is defined as the lowest concentration which suppresses the growth of bacteria. The experiment was repeated 5 times for *M. luteus*; 2 times for *M. smegmatis* and 3 times for *M. tuberculosis*. MICs for *M. luteus* and *M. smegmatis* were determined during 24 hours; for *M. tuberculosis* – 8 days.

KI_50_ was examined for RpfSm 5 times and 3 times for _ΔDUF_RpfB. ND – not done. NI – no inhibition was observed.

### The Influence of NPT on the Secondary Structure of Rpf

To exclude possible alterations of the Rpf secondary structure caused by NPT, CD measurement studies were undertaken using both Rpfs. Figure S2 A, B shows that the addition of the inhibitor (VII) did not produce significant changes in the CD spectra.

### Interaction of NPT Compounds with Rpf (Fluorescence Quenching Analysis)

**Figure 2:**
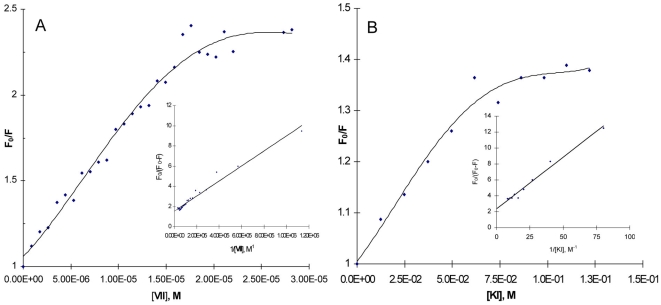
Fluorescence quenching of _ΔDUF_Rpf B. A. quenching by (VII). _ΔDUF_Rpf B was dissolved in 50 mM phosphate buffer pH 7.5 at concentration 40 µg/ml. Results were shown in the Stern–Volmer plot and in the modified Stern–Volmer plot (insert). Excitation - 280 nm, fluorescence intensity was recordered at 340 nm. Experiment was performed 2 times, with similar results. One experiment is shown. B. quenching by KI. For details see legend to Fig. 2**A**. Experiment was performed 2 times, with similar results. One experiment is shown.

The existence of several tryptophanes and phenylalanines residues in the Rpf molecule enables one to analyse an interaction between NPTs and Rpf by fluorescence quenching. The addition of the highly efficient inhibitor of the enzymatic activity of Rpf, the compound (VII), to _ΔDUF_RpfB resulted in significant quenching of its auto-fluorescence (excitation 280 and emission 340) (Figure 2A). Application of the standard Stern-Volmer equation, (1) clearly showed a heterogeneous type of quenching with different accessibility of tryptophan residues for the inhibitor. In this case, we applied a modified form of the Stern-Volmer equation (2) where *f_a_* is an accessible fraction of fluorochromes. From equation (2), the accessible fraction of _ΔDUF_RpfB tryptophanes for compound (VII) (*f_a_*) was close to 65%. These results were compared with quenching of _ΔDUF_RpfB by iodide which interacts with the exposed fluorochromes only [26]. Similar to the NPT compounds, iodide produced the heterogeneous type of quenching (Figure 2B); however, the accessible fraction in this case was estimated close to 40%. Because the _ΔDUF_RpfB molecule contains 5 tryptophan residues (the major contributors in an auto-fluorescence under excitation used), 65% of accessibility to (VII) corresponds to three tryptophanes. 40% accessibility for iodide corresponds two exposed tryptophan residues localized on the molecule surface, indicating that the substance (VII) interacted with at least two exposed and one buried tryptophan. Note, that in case of inactive compound (VIII) the accessible fraction was similar to iodide (not shown).

### Biological Activity of the NPT Compounds

The biological effect of the NTP compounds was studied in three different Rpf-producing bacteria, such as *M. luteus, M. smegmatis* and *M. tuberculosis*. *M. luteus*, containing the single *rpf* gene essential for growth [29], is an excellent model organism for study the inhibitors of Rpf. Seven out of ten tested compounds inhibited *M. luteus* growth in the NB liquid medium (MICs were between 1-10 µg/ml). Moreover, there was clear correlation between inhibition of the enzymatic activity of Rpf and bacteriostatic effects displayed by the compounds. For example, the compounds (II) and (VIII) did not influence the Rpf enzymatic activity and had no effect on *M. luteus* growth (Table 1).

However, the main known function of Rpfs is resuscitation of dormant or “non-culturable” cells [15], [20]. Therefore, we tested the effect of NPT on resuscitation of dormant mycobacteria in two dormancy models, which we had previously developed.

**Figure 3:**
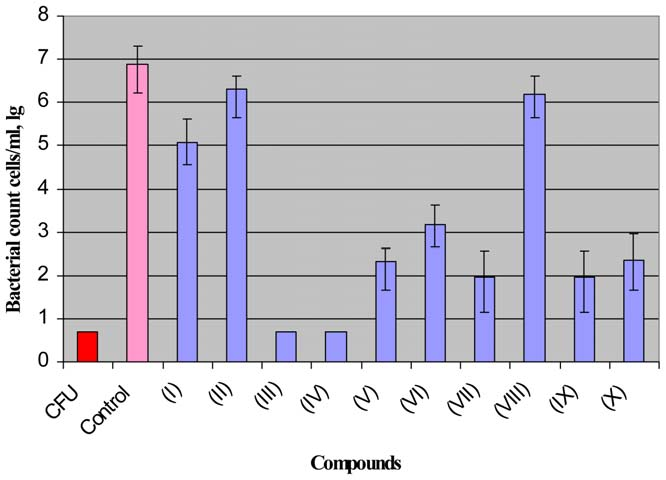
Influence of NPT compounds on resuscitation of *M. smegmatis* “non-culturable” cells. Resuscitation was performed by the MPN assay (for details see “Materials and methods”). Inhibitors were added at 1 µg/ml to some wells. Plates were incubated for 2 weeks at 37°C with shaking, 100 rpm. CFU of the culture before resuscitation was <5 cells/ml. Bars represent (95%) confidence limits for MPN assay. This experiment was repeated six times using different starved cultures with similar results. One typical experiment is shown.

**Figure 4:**
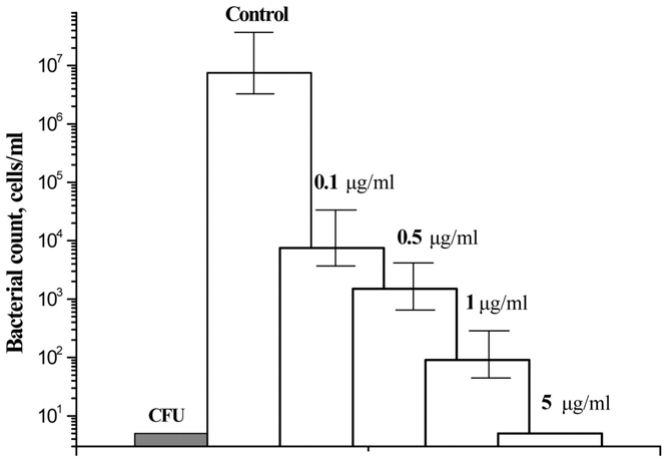
Influence of compound (IX) on resuscitation of “non-culturable” cells of *M. smegmatis*. Resuscitation of “non-culturable” cells of *M. smegmatis* obtained in the stationary phase (see “Materials and methods”) was performed in the liquid medium by MPN assay as described in the “Materials and methods”. Plates with resuscitating cells were incubated for 2 weeks at 37°C with shaking at 100 rpm. Filled column – CFU count before resuscitation. Opened columns – MPN counts. Concentrations of compound (IX) added to the resuscitation medium are shown above each column. Bars represent (95%) confidence limits for MPN and ±SD for CFU.

**Figure 5:**
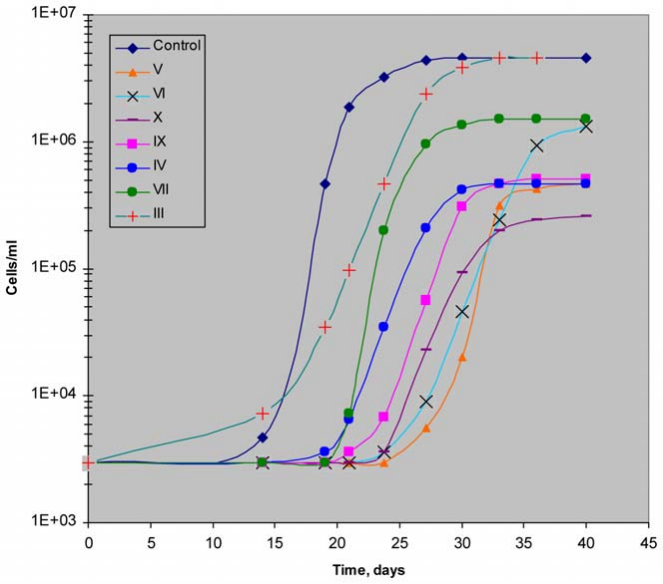
Influence of NPT compounds on resuscitation of *M. tuberculosis* “non-culturable” cells obtained *in vitro*. “Non-culturable” cells of *M. tuberculosis* cultures were obtained after cell cultivation during 50 days in Sauton's medium without K^+^. Resuscitation was performed in the absence or presence of inhibitors at 10 µg/ml using 10-fold serial dilutions (MPN assay). MPN values were counted periodically during resuscitation time until their stabilization.

In the first model the majority of *M. smegmatis* cells lose the ability to produce colonies on agar or grow in a liquid medium upon incubation in prolonged stationary phase [30]. Such “non-culturable” cells could be resuscitated in a liquid medium when Rpfs added exogenously or overexpressed from a high-copy number plasmid [20], [21]. A Most Probable Number assay was applied to estimate the number of resuscitated cells. We consider resuscitation successful when the ratio MPN/CFU is equal or more than 1000. As follows from Figure 3 the addition of any NTP compound, (except (I), (II) and (VIII)), significantly suppressed resuscitation of *M. smegmatis* cells. The observed inhibitory effect was concentration dependent (Figure 4), and the most potent compounds (III, IV, VII, IX) were active at 0.1–1 µg/ml concentrations which did not inhibit growth of active cells of *M. smegmatis* ( not shown). In the second model “non-culturable” cells of *M.tuberculosis* resuscitate spontaneously, nevertheless this process is Rpf-dependent since the Rpf mutants were unable to resuscitate [6]. We found that compound (IX) and its derivatives (V-VII and X) as well as compound (IV) at concentration 10 µg/ml increased time which need for developing of visible growth in the MPN assay and decreased the final values of MPN in comparison with untreated resuscitating cells (Figure 5, Table S1). However, one of the most potent compounds for inhibition of resuscitation of *M.smegmatis* (III) practically did not influence resuscitation of *M.tuberculosis*. Compounds (I,VIII) had no influence on the resuscitation kinetics (not shown). None of the compounds tested suppressed growth of active cells of *M. tuberculosis* at the same concentration (not shown).

## Discussion

The structural studies of the conserved domain of Rpf could be considered as a “compact hybrid” of c-type lysozyme and lytic transglycosylases [13], [14], two classes of enzymes cleaving 1, 4-β-glycosidic bond between N-acetylmuramic acid and N-acetylglucosamine in peptidoglycan. Two inhibitors of lytic transglycosylases from Gram-negative bacteria (bulgecine and NAG-thiazoline) were reported, but they have never been tested against Gram-positive bacteria [31], [32]. Therefore, a novel class of NPT inhibitors of the muralytic activity of Rpf opens a new way in the study of cell-wall hydrolyzing enzymes from Gram-positive bacteria, in particular, mycobacteria.

Through our efforts related to the search of new antimicrobial compounds we observed that derivatives of ortho-nitrodialkyldithiocarbamate have potent antimycobacterial activity [33]. As a result of different chemical modifications of this basic structure we replaced the dialkyldithiocarbamate group by a thiocyanate group which has similar electron affinity. Using a facile synthesis of the target we obtained several ortho nitroarylthiocyanate derivatives (Figure 1), based on the reaction of nucleophylic substitution, the active chloroatom in nitroaryl moiety was substituted by thiocyanate ion.

We found that some of these compounds strongly suppressed germination of fungal spores (unpublished). Structural similarity between Rpf and cell wall lytic enzymes involved in spore germination (see above) allowed us to suggest that NPTs may influence on the activity of Rpf.

We first showed that most of candidate compounds indeed inhibit muralytic activity of Rpf in the non-competitive mode (Figure S1). The measurements of the fluorescence quenching unequivocally prove a strong interaction between inhibitory NPTs and the internal region of Rpf, providing a molecular basis for inhibition of the enzymatic activity. Based on the published crystal structure of _ΔDUF_RpfB [14], we suggest that the compound (VII) is able to interact with the exposed Trp residues, 349, 285 and with one out of three buried Trp-297,316 and 352. Because buried Trp-316 and Trp-297 are localized closely, their accessibility to the quencher would be similar, therefore we could suggest that compound (VII) interacts not with these two residues but with Trp-352 which lies in the catalytic site of the molecule [13]. From this, interaction of the inhibitor (VII) with the catalytic site appears plausible. The absence of significant changes in the secondary structure of Rpf (Figure S2) ruled out a possible non-specific inactivation of the enzyme, for example, protein aggregation, under NPT binding. Specificity of NPT as inhibitors of Rpf enzyme remains to be proved however, NPT did not influence the activity of some well-known enzymes as alkoholdehydrogenase (1.1.1.1), laccase (1.10.3.2) and trypsin (3.4.21.4) (Table S2). The observed inhibitory effect of NPTs on growth of *M. luteus* is in a good accordance of Rpf essentiality in this bacterium. Remarkably, we found a good correlation between the effectiveness of different NPTs to inhibit the enzymatic activity of Rpf and to arrest growth of *M. luteus* cells (with the exception of compound (I)). The inhibitory concentration range for these compounds was also similar for both processes.

It was stated that Rpf proteins stimulate growth of cells at suboptimal or stressful conditions, but not of actively multiplying cells [24]. In particular, the deletion of *rpf* genes did not significantly impair the growth of the axenic cultures of *M. tuberculosis*, but exhibited a vital effect on their resuscitation in the liquid medium *in vitro* [5], [6] or an activation of chronic infection *in vivo* [8]. According to this, NPT were active against resuscitation of “non-culturable” cells of both *M. smegmatis* and *M.tuberculosis* but to the less extent against the growth of viable cells, providing additional evidence for collective non-essentiality of *rpf* genes in *M.tuberculosis* [6]. Thus, on the basis of data obtained from several experimental models we may conclude that 2-nitrophenylthiocyanates represent a first group of inhibitors of the Rpf-mediated mycobacterium resuscitation.

Availability of derivatives of NPT with different effects against Rpf activities provides some insight in the mechanism of their action. So, the failure of the compound (VIII) to inhibit 4-MUF-3-NAG hydrolyzing activity, growth of *M. luteus* and resuscitation of *M. smegmatis*, most likely could be explained by presence the negative charge on the molecule. Indeed, in published crystal structure of _ΔDUF_RpfB, a negatively charged region in the catalytic site has been established [14]. This may produce a significant obstacle for interaction of the compound (VIII) with the catalytic site. Indeed, according to the fluorescence studies, (VIII) doesn't interact with the buried tryptophanes.

Despite a rather good correlation between anti-enzymatic and anti-resuscitation activities observed in this study, more experiments are required to confirm that Rpf proteins are, indeed, the targets for NPT in cells. The unique ability of NPT to suppress or at least delay resuscitation of “non-culturable” mycobacteria makes them attractive agents for specific target - non-replicating mycobacteria.

Because Rpf proteins are likely important for the establishing of chronic [7], [8] and even probably acute [5] form of experimental (mice) tuberculosis, such compounds could be prospective for preventing of the latent tuberculosis activation or could be efficient against active TB. The task of the future is to study an activity of the NPT compounds in the animal experimental tuberculosis models.

## Supporting Information

Figure S1 Inhibition of 4-MUF-3-NAG hydrolysis by RpfSm in the presence of (**VII**). 4-MUF-3-NAG hydrolysis by RpfSm (30 µg/ml) was performed according to “Materials and methods” without inhibitor (♦) or in the presence of (**VII**): - 0.5 µg/ml; ▴ - 1 µg/ml; × - 5 µg/ml; *- 10 µg/ml. The Lineweaver-Burk plot
(4.81 MB TIF)

Figure S2 Influence of compound (VII) on the secondary structure of Rpfs. CD spectra of ΔDUFRpf B (A) and Rpf Sm (B) in absence and presence of (VII) were obtained as described in the “Materials and methods”. Concentration of Rpf Sm was 40 µg/ml and ΔDUFRpf B- 60 µg/ml. Concentration of (VII) were 2 and 4 µg/ml. Inhibitor was dissolved in acetonitrile. The spectra were recorded from 195 to 260 nm and the average of three wavelength scans is presented.
(5.81 MB TIF)

Table S1 Influence of NPT compounds on resuscitation of *M. tuberculosis* “non-culturable” cells obtained *in vitro*. Confidence limits (95%) for MPN are shown. These numbers present logarithmic transformation of cell concentration (cells/ml) estimated by MPN assay. The “lower “ and “upper” values are logarithmic transformation of cell concentrations representing 95% confidence limits of the assay.
(0.04 MB DOC)

Table S2 Influence of compounds (III) and (VII) on activity of some enzymes τ_1/2_- time is needed to achieve a 50% level of substrate conversion. *Alcoholdehydrohenase(1.1.1.1) activity*: ADH was dissolved in 50 mM phospahe buffer pH 7 at the concentration 10 µg/ml. The rate of the reaction was judged by transformation of NAD^+^ (1 mM) into NADH+H^+^. As the substrate ethanol was used. Activity was measured by OD_340_. *Laccase(1.10.3.2) activity:* Laccase was dissolved in the 50 mM citrate-phosphate buffer, pH4.5 at concentration 9 µg/ml. Pyrocatechin was used at concentration 11 µg/ml. Activity was measured by OD_410_. *Trypsin(3.4.21.4) activity*: The enzyme at concentration 8.0 µg/ml was dissolved in 50 mM Tris-HCl buffer pH 8.0. Concentration of the substrate (N-CBz-Gly-Gly-Arg-β-naphthylamide - Sigma-Aldrich)- 11 µg/ml. Registration of the kinetic curves was made fluorimetrically - excitation wavelength - 330 nm; emission - 420 nm.
(0.02 MB DOC)
