# Supplementary figures and images for: Finding of the Low Molecular Weight Inhibitors of Resuscitation Promoting Factor Enzymatic and Resuscitation Activity
Source: PLoS One. 2009 Dec 16;4(12):e8174. doi: 10.1371/journal.pone.0008174 (PMC2790607; doi:10.1371/journal.pone.0008174)

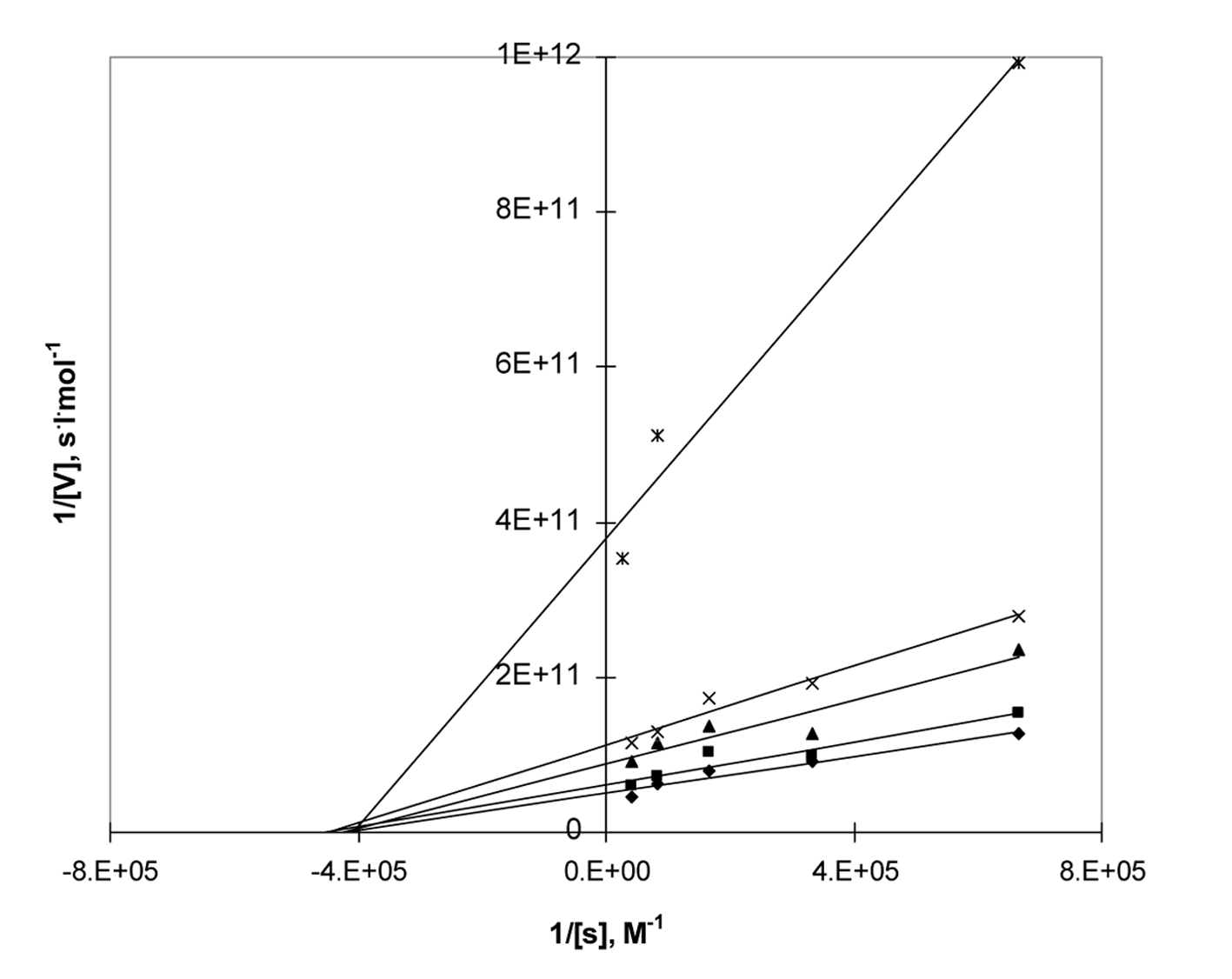

Supplement: Figure S1 — Inhibition of 4-MUF-3-NAG hydrolysis by RpfSm in the presence of (VII). 4-MUF-3-NAG hydrolysis by RpfSm (30 µg/ml) was performed according to “Materials and methods” without inhibitor (♦) or in the presence of (VII): - 0.5 µg/ml; ▴ - 1 µg/ml; × - 5 µg/ml; *- 10 µg/ml. The Lineweaver-Burk plot (4.81 MB TIF) [file pone.0008174.s001.tif]

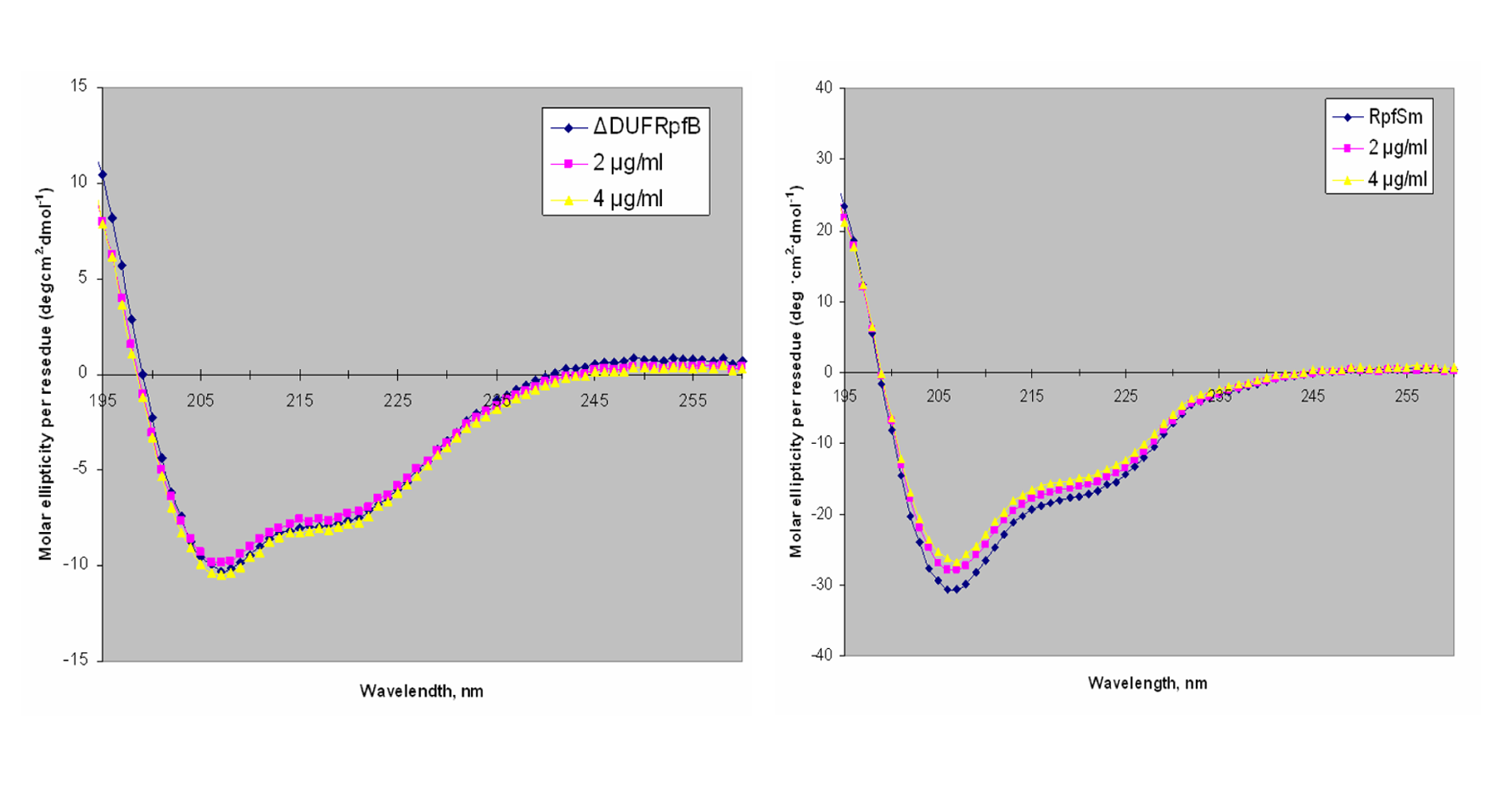

Supplement: Figure S2 — Influence of compound (VII) on the secondary structure of Rpfs. CD spectra of ΔDUFRpf B (A) and Rpf Sm (B) in absence and presence of (VII) were obtained as described in the “Materials and methods”. Concentration of Rpf Sm was 40 µg/ml and ΔDUFRpf B- 60 µg/ml. Concentration of (VII) were 2 and 4 µg/ml. Inhibitor was dissolved in acetonitrile. The spectra were recorded from 195 to 260 nm and the average of three wavelength scans is presented. (5.81 MB TIF) [file pone.0008174.s002.tif]
