# Supplementary material for: Finding of the Low Molecular Weight Inhibitors of Resuscitation Promoting Factor Enzymatic and Resuscitation Activity
Source: PLoS One. 2009 Dec 16;4(12):e8174. doi: 10.1371/journal.pone.0008174 (PMC2790607; doi:10.1371/journal.pone.0008174)
Supplement: Table S1 — Influence of NPT compounds on resuscitation of M. tuberculosis “non-culturable” cells obtained in vitro. Confidence limits (95%) for MPN are shown. These numbers present logarithmic transformation of cell concentration (cells/ml) estimated by MPN assay. The “lower “ and “upper” values are logarithmic transformation of cell concentrations representing 95% confidence limits of the assay. (0.04 MB DOC) [file pone.0008174.s003.doc]

| **Time,days** | **Control** | | | (**IX**) | | |  | (**V**) | | (III) | | |
| --- | --- | --- | --- | --- | --- | --- | --- | --- | --- | --- | --- | --- |
|  |  | Low | High |  | Low | High |  | Low | High |  | Low | High |
| **0** | 3.48 | 0.00 | 3.98 | 3.48 | 0.00 | 3.98 | 3.48 | 0.00 | 3.98 | 3.48 | 0.00 | 3.98 |
| **14** | 3.48 | 0.00 | 3.98 | 3.48 | 0.00 | 3.98 | 3.48 | 3.48 | 3.48 | 3.48 | 0.00 | 3.98 |
| **19** | 5.66 | 4.95 | 6.30 | 3.48 | 0.00 | 3.98 | 3.48 | 3.48 | 3.48 | 3.86 | 3.11 | 4.3 |
| **21** | 6.38 | 5.62 | 7.00 | 3.56 | 2.23 | 4.26 | 3.48 | 3.48 | 3.48 | 4.63 | 3.95 | 5.26 |
| **23** | 6.38 | 5.62 | 7.00 | 3.96 | 3.15 | 4.58 | 3.48 | 3.48 | 3.48 | 4.63 | 3.95 | 5.26 |
| **27** | 6.66 | 5.95 | 7.30 | 4.32 | 2.65 | 4.62 | 3.86 | 3.11 | 4.26 | 5.66 | 4.95 | 6.30 |
| **30** | 6.66 | 5.95 | 7.30 | 5.66 | 4.95 | 6.30 | 4.30 | 3.65 | 4.62 | 6.38 | 5.62 | 7.00 |
| **33** | 6.66 | 5.95 | 7.30 | 5.66 | 5.66 | 5.66 | 5.66 | 4.95 | 6.30 | 6.38 | 5.62 | 7.00 |
| **36** | 6.66 | 5.95 | 7.30 | 5.66 | 5.66 | 5.66 | 5.66 | 5.66 | 5.66 | 6.38 | 5.62 | 7.00 |
| **40** | 6.66 | 5.95 | 7.30 | 5.66 | 5.66 | 5.66 | 5.66 | 5.66 | 5.66 | 6.38 | 5.62 | 7.00 |
| **Time,days** | (**VI**) | | | (**X**) | | |  | (**VII**) | | (**IV**) | | |
|  |  | Low | High |  | Low | High |  | Low | High |  | Low | High |
| **0** | 3.48 | 0.00 | 3.98 | 3.48 | 0.00 | 3.98 | 3.48 | 0.00 | 3.98 | 3.48 | 0.00 | 3.98 |
| **14** | 3.48 | 3.48 | 3.48 | 3.48 | 3.48 | 3.48 | 3.48 | 3.48 | 3.48 | 3.48 | 0.00 | 3.98 |
| **19** | 3.48 | 3.48 | 3.48 | 3.48 | 3.48 | 3.48 | 3.48 | 3.48 | 3.48 | 3.56 | 2.23 | 4.26 |
| **21** | 3.48 | 3.48 | 3.48 | 3.48 | 3.48 | 3.48 | 3.86 | 3.11 | 4.26 | 3.96 | 3.15 | 4.58 |
| **23** | 3.56 | 2.23 | 4.26 | 3.48 | 3.48 | 3.48 | 5.38 | 4.62 | 6.00 | 4.36 | 3.66 | 4.97 |
| **27** | 3.96 | 3.15 | 4.58 | 4.36 | 3.66 | 4.97 | 6.18 | 5.57 | 6.62 | 5.38 | 4.62 | 6.00 |
| **30** | 4.66 | 3.95 | 5.30 | 4.97 | 4.26 | 5.62 | 6.18 | 6.18 | 6.18 | 5.66 | 4.95 | 6.30 |
| **33** | 5.38 | 4.62 | 6.00 | 5.38 | 4.62 | 6.00 | 6.18 | 6.18 | 6.18 | 5.66 | 4.95 | 6.30 |
| **36** | 5.97 | 5.26 | 6.62 | 5.38 | 5.38 | 5.38 | 6.18 | 6.18 | 6.18 | 5.66 | 4.95 | 6.30 |
| **40** | 6.18 | 5.57 | 6.62 | 5.38 | 5.38 | 5.38 | 6.18 | 6.18 | 6.18 | 5.66 | 4.95 | 6.30 |
