# Supplementary material for: Finding of the Low Molecular Weight Inhibitors of Resuscitation Promoting Factor Enzymatic and Resuscitation Activity
Source: PLoS One. 2009 Dec 16;4(12):e8174. doi: 10.1371/journal.pone.0008174 (PMC2790607; doi:10.1371/journal.pone.0008174)
Supplement: Table S2 — Influence of compounds (III) and (VII) on activity of some enzymes τ1/2- time is needed to achieve a 50% level of substrate conversion. Alcoholdehydrohenase(1.1.1.1) activity: ADH was dissolved in 50 mM phospahe buffer pH 7 at the concentration 10 µg/ml. The rate of the reaction was judged by transformation of NAD+ (1 mM) into NADH+H+. As the substrate ethanol was used. Activity was measured by OD340. Laccase(1.10.3.2) activity: Laccase was dissolved in the 50 mM citrate-phosphate buffer, pH4.5 at concentration 9 µg/ml. Pyrocatechin was used at concentration 11 µg/ml. Activity was measured by OD410. Trypsin(3.4.21.4) activity: The enzyme at concentration 8.0 µg/ml was dissolved in 50 mM Tris-HCl buffer pH 8.0. Concentration of the substrate (N-CBz-Gly-Gly-Arg-β-naphthylamide - Sigma-Aldrich)- 11 µg/ml. Registration of the kinetic curves was made fluorimetrically - excitation wavelength - 330 nm; emission - 420 nm. (0.02 MB DOC) [file pone.0008174.s004.doc]

| Enzyme | Control τ_1/2_,s | (III) τ_1/2_,s | | (VII) τ_1/2_,s | |
| --- | --- | --- | --- | --- | --- |
|  |  | 4 µg/ml | 10 µg/ml | 4 µg/ml | 10 µg/ml |
| Alchoholdehydrohenase | 1.7 | 1.7 | 1.7 | 1.75 | 1.7 |
| Laccase | 3.6 | 3.6 | 3.5 | 3.6 | 3.5 |
| Trypsin | 10 | 10 | 10 | 10 | 10 |
